# Recent Advances on Queen Bee Larvae: Sources, Chemical Composition, and Health-Benefit Bioactivities

**DOI:** 10.3390/foods15010089

**Published:** 2025-12-28

**Authors:** Pengbo Liang, Xinyu Yu, Meifei Zhu, Bin Yuan, Shanshan Li, Fuliang Hu

**Affiliations:** College of Animal Sciences, Zhejiang University, Hangzhou 310058, China; 22417024@zju.edu.cn (P.L.); yuxinyu@zju.edu.cn (X.Y.); meifeizhu@zju.edu.cn (M.Z.); yuan_bin322@zju.edu.cn (B.Y.)

**Keywords:** queen bee larvae, nutritional composition, anti-aging, anti-inflammatory, anti-oxidant properties

## Abstract

Queen bee larvae, a by-product of royal jelly production, represents a largely underutilized yet promising economic resource. Numerous studies have shown that these larvae are rich in proteins, amino acids, fatty acids, and minerals, indicating high nutritional value. Research has also highlighted their bioactivities, including anti-aging, anti-inflammatory, and antioxidant properties. This review summarizes the current knowledge on the nutritional composition and biological functions of queen bee larvae, providing a scientific foundation for their comprehensive utilization. The findings support their potential applications in food science and health-related industries.

## 1. Introduction

Global food demand is expected to reach 210% of the 2005 level by 2050, with protein requirements in major developing countries projected to increase by 76% [1]. Under such pressure, many insects have attracted attention due to their high protein content and excellent quality. Insects represent a promising food resource due to the high farming efficiency, environmental adaptability, sustainable land use, and low greenhouse gas emissions associated with their production [2,3,4,5,6,7,8]. With over one million insect species globally, approximately 2205 are recognized as edible [8]. Among these, the honeybee is an edible insect that has been successfully reared under controlled human cultivation. Honeybees not only provide traditional hive products such as honey, royal jelly (RJ), and bee pollen but also represent a direct source of nutrition through their larvae [9].

China has a history of insect consumption dating back more than 2000 years, with bee larvae and pupae remaining integral to the culinary traditions of various ethnic groups [10,11,12]. In regions such as Yunnan, QBL and pupae are traditionally consumed and form part of the local cultural heritage [10,11,12,13,14,15]. Therefore, QBL possess immense social and economic potential [12]. QBL, as a byproduct of RJ production, are produced alongside RJ during the peak flowering season from March to September each year. During this period, a standard *Apis mellifera* hive can yield 5000–12,000 g RJ and 1000–2400 g QBL [16,17]. According to a report by the Chinese Academy of Agricultural Sciences, China’s annual RJ production averages around 3000 tons (https://iar.caas.cn//kwxh/zgfy/fyjj/aed0b2a3e2ed44c0bd659f6d9763e5d9.htm (accessed on 8 September 2025)), yielding an estimated 600 tons of QBL each year [16,17].

Bee larvae and pupae are notably rich in proteins, essential amino acids, unsaturated fatty acids, minerals, and trace elements [10,12,17,18,19,20,21,22]. They also exert bioactivity, including anti-aging, anti-inflammatory, and antioxidant properties [21,22,23,24]. Beyond this, the safety of QBL is supported by recent studies. For example, Tong et al. (2024) conducted a sub-chronic oral toxicity study in mice given freeze-dried queen bee larva powder (QBLP) up to 5 g/kg/day and observed no treatment-related mortality, clinical abnormalities, systemic toxicity, or histopathological lesions in major organs [25]. Consistently, Thuraphan et al. (2024) reported that QBL protein extract did not induce skin irritation, using hen egg test-chorioallantoic membrane assay at the concentration of 10 mg/mL [23]. These findings support a favorable safety profile for QBL and the associated extracts within the tested dose ranges. QBL represent the early developmental stage of the queen bee. Sharing the same genetic background as worker bees, QBL are essential in inducing adult worker bees to secrete RJ and comprise an inevitable byproduct of the RJ production process [26,27]. Despite the recognized nutritional value, the chemical composition and biological activities of QBL remain insufficiently studied. By integrating current knowledge, we seek to evaluate its potential as a functional food that may contribute to human health and address future food security challenges.

## 2. Methodology

The literature search focused on studies related to QBL, with particular emphasis on reports concerning its sources and production, chemical composition, and biological activity. The following databases were searched: Google Scholar, Web of Science, Wanfang Data, PubMed, Scopus, and the China National Knowledge Infrastructure (CNKI). The most recent search was conducted in September 2025, incorporating records published between 1997 and 2025. The retrieval strategy involved combining QBL with specific subject terms. Examples include (‘queen bee larva’, ‘queen larvae’, ‘queen brood’, ‘Apis mellifera larva’, ‘queen bee larvae powder’, QBL, and QBLP), whilst equivalent Chinese keywords were employed for CNKI. If the literature studies identified in the preliminary search met all of the following criteria, they were considered for inclusion: (i) the research subjects involve QBL (typically aged 1–5 days) or materials derived from QBL (such as QBL powder, QBL protein extracts, QBL hydrolysates); (ii) the study reports at least one outcome related to QBL’s chemical composition (e.g., proteins, amino acids, fatty acids, minerals), biological activity (e.g., anti-oxidant, anti-aging, metabolic regulation effects), processing characteristics, or safety; (iii) the study provides production details and biological characterization results for QBL. Peer-reviewed journal articles were prioritized; however, theses or conference papers containing QBL-related data were also considered. Studies were excluded if (i) the study focuses on other apicultural products (e.g., royal jelly) without relevant QBL data; (ii) the study explicitly targets worker or drone larvae/pupae rather than all bee larvae; (iii) patent documents are unavailable or lack extractable information; (iv) full-text access remains unattainable after multi-platform searches. Given the specific scope of this review (studies focusing on QBL or QBL-derived materials with extractable data on composition, bioactivity, processing, or safety), the number of eligible publications remained limited; therefore, 59 references were included in the final reference list.

## 3. Sources of QBL

**Figure 1:**
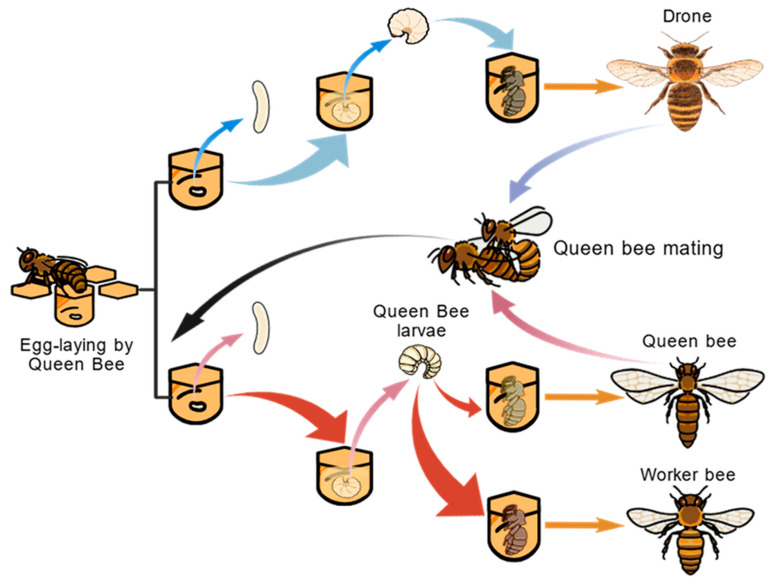
Developmental trajectories of *Apis mellifera* castes: caste differentiation mediated by larval nutrition and cell morphology [28].

The developmental cycle of honeybees comprises four stages—egg, larva, pupa, and adult—as shown in Figure 1 [28]. A typical honeybee colony consists of three castes, including queen, workers, and drones, each exhibiting distinct developmental duration, nutritional requirements, behavioral characteristics, and functional roles [29,30,31,32,33,34,35,36]. The queen bee mates with drones to produce diploid, fertilized eggs, which hatch into female larvae after a three-day incubation period. Worker bees feed these larvae with RJ, a secretion from their hypopharyngeal and mandibular glands. While worker-destined larvae receive RJ only during the first three days, queen-destined larvae are exclusively fed RJ throughout their entire development. This differential feeding strategy is critically exploited in apiculture for RJ production [26,37,38]. QBL represent the early developmental stage of the reproductive caste in *Apis mellifera*. Although genetically identical to worker larvae, QBL follow a distinct caste-differentiation pathway, driven by continuous RJ feeding [26,27]. During the 1–5 day larval period, QBL undergo rapid growth and exhibit high metabolic activity [26,39]. These biological characteristics are essential for understanding their nutritional composition and bioactivities.

**Figure 2:**
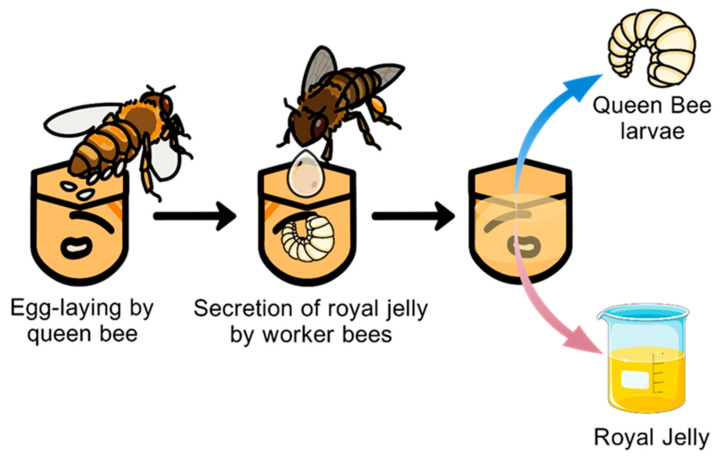
The production process of QBL (*Apis mellifera*).

Specifically, QBL, which are defined as diploid female larvae aged 1 to 5 days, are a direct byproduct of RJ harvesting. Generally, female larvae less than one day old are transferred from the beehive to artificial queen cells to simulate natural queen cell rearing conditions. This setup stimulates worker bees to secrete RJ into the queen cells. After larval grafting, the primary harvesting of royal jelly typically occurs approximately 72 h (3 days) later and is collected using fine forceps, after which the accumulated RJ is harvested using a small spoon [16,26,37,38,40,41]. Then, the queen cells are cleaned and can be re-grafted with new larvae to start the next production cycle. During this process, dead female larvae cannot stimulate worker bees to produce RJ. Therefore it is necessary to extract the healthy QBL for use. Additionally, a single hive can produce approximately 400 g of RJ under conditions of fixed-location beekeeping in Zhejiang Province using the Zhejiang RJ bee during the spring season [16]. The QBL, as organisms that stimulate worker bees to secrete RJ, represents an inevitable byproduct of the entire RJ production process (Figure 2) [16,26,42,43].

## 4. Chemical Composition of QBL

### 4.1. Proteins

QBL represents a high-quality protein source. The protein content of QBL shows notable variability across studies, largely attributable to differences in larval age, extraction conditions, and nitrogen-to-protein conversion factors [12,21,23,25]. On a dry weight basis, crude protein ranges from 41.50% to 55.8% (Kp6.25) [10,12,17,21,25], with amino acids constituting 37.30–47.57% of dry matter [44,45]. Essential amino acids account for approximately 49% of total amino acids, with ratios of EAA (essential amino acids)/NEAA (non-essential amino acids) meeting FAO/WHO standards for high-quality protein [12,45].

As shown in Table 1, alkaline extraction at pH 12.57 yielded a protein extraction rate of 48.7, though alternative methods using a lower pH of 10.0 and revised nitrogen-to-protein conversion factors (e.g., 5.6 for larvae) reported lower crude protein values, e.g., 22.1 and 19.0 [30,31]. This difference is attributed to milder alkaline conditions, which could reduce protein solubilization. Furthermore, the use of an insect-specific conversion factor excludes contributions from substantial non-protein nitrogen components (such as chitin-bound nitrogen and nitrogenous metabolites) [23,30,46]. Ultrasound-assisted extraction improved efficiency to 30.08% [31]. The primary limiting amino acids are methionine and threonine [45] (Table 2).

### 4.2. Lipids

**Figure 3:**
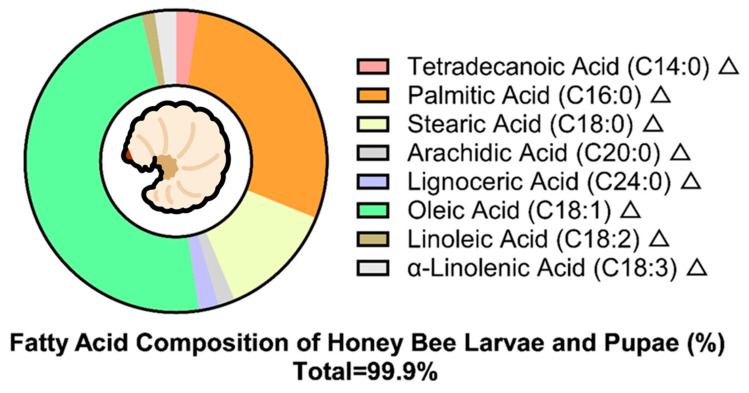
Fatty acid composition (% of total lipids) in *Apis mellifera* larvae and pupae. Note: Total quantified lipids account for 99.9% of detectable fatty acids.

The crude fat content of QBL ranges from 15.0% to 28.1% on a dry weight basis [10,30,49], while the lipid content constitutes 3.43% of fresh weight and is slightly higher than that of RJ (3.06%) [50]. The predominant lipid components were identified as triglycerides (797.5 mg/g lipid) and phospholipids (89.9 mg/g lipid). The differences in lipid content are mainly due to the different nutritional strategy for QBL [10,28]. Fatty acid profiles are dominated by oleic acid (C18:1), comprising 45.9–46.6% of total fatty acids. Other major fatty acids include palmitic acid (34.5%) and stearic acid (10.4%) [30]. Furthermore, as shown in Figure 3, the presence of derosterol, Δ7—stigmasterol, and cycloartenol has been identified in QBL [50].

### 4.3. Carbohydrates

The total carbohydrate content of QBL has been reported to range from 11.6% to 54.1% in freeze-dried larvae powder [10,30,31]. The considerable variability arises largely from differences in how chitin is treated analytically. Chitin is a nitrogen-containing polysaccharide composed of N-acetylglucosamine units. Therefore, some studies incorporate the entirety of chitin within total carbohydrates, while others account for its nitrogen contribution separately, as part of the non-protein nitrogen fraction, when using nitrogen-based quantification methods (e.g., the Kjeldahl assay). These methodological differences lead to substantial variation in the reported carbohydrate content of QBL [46]. The predominant sugars are glucose and fructose, which account for 6.7% and 3.4% of the total carbohydrates, respectively. Additionally, the non-reducing sugar trehalose is an important component, comprising 41.4% of the total sugars [44,47].

### 4.4. Other Components

QBL contain notable amounts of B vitamins, with reported concentrations of vitamin B1 (2.97 mg/100 g of dry weight), B2 (3.00 mg/100 g of dry weight), B6 (1.91 mg/100 g of dry weight), and niacin (29.14 mg/100 g of dry weight) [17]. Their mineral composition includes both macro- and micro-elements, such as potassium, calcium, magnesium, copper, iron, manganese, and zinc [17,22,31]. The analysis of mineral composition revealed that QBL (*Apis mellifera*) contained significantly elevated levels of calcium (Ca), magnesium (Mg), copper (Cu), iron (Fe), and manganese (Mn) compared to other commonly consumed meats. In contrast, the potassium (K) content was lower only than that of beef sirloin and chicken breast, while its zinc (Zn) content ranked below mutton leg and beef sirloin. Notably, QBL exhibited the lowest sodium (Na) concentration among all meat types analyzed [12].

## 5. Health Benefit Effects of QBL

The preliminary investigation into the biological activities of QBL dates back to the last century. Since the beginning of the 21st century, and particularly over the past decade, there has been a significant increase in research output in this field. This section provides a comprehensive overview of the reported biological activities of QBL, which include anti-aging, anti-oxidant, metabolic regulation properties, among others (Table 3).

### 5.1. Anti-Aging Effects

QBL and their derivatives have attracted growing scientific interest due to their notable anti-aging properties, as evidenced by multiple experimental models. Early work by Ni et al. demonstrated that QBL supplementation significantly extended the mean lifespan of male houseflies (*Musca domestica*) and improved anti-stress capacity in NIH mice [55]. The incorporation of QBL at concentrations of 5–20% into a milk powder-based diet led to a substantial increase in lifespan. Furthermore, even under D-galactose-induced stress conditions, male houseflies fed with 40% QBL also exhibited a significantly prolonged average lifespan. These findings suggest that QBL may exert its life-extending effects by enhanced scavenging of free radicals and modulating of the anti-oxidant system [55]. More recent studies further support these findings and provide deeper mechanistic insights. Using *Caenorhabditis elegans* as a model, Tong et al. (2022) [24] reported that QBLP extended mean lifespan by up to 28.1% and improved healthspan in a dose-dependent manner. Transcriptomic analysis indicated upregulation of longevity-associated genes (*sod-3, hsp-12.6, lips-7,* and *lips-17*) and, crucially, lifespan extension was abolished in dauer formation allele (DAF)-16 mutant strains, confirming the essential role of the DAF-16 pathway in mediating anti-aging effects.

Moreover, QBLP has demonstrated significant anti-aging effects in D-galactose-induced aging mice. QBLP administration improved cognitive performance and spatial memory, increased organ indices (including the brain, heart, kidney, liver, spleen), reduced brain cell necrosis, decreased serum malondialdehyde (MDA) levels, and elevated skin moisture. Additionally, QBLP enhanced gut microbiota diversity, particularly elevating beneficial families such as *Ruminococcaceae* and *Lachnospiraceae*, and through the synergistic modulation of a core metabolic network involving glutamate, alanine, proline, and aspartate, QBLP ameliorated the D-galactose-induced dysregulation of the serum amino acid profile, ultimately mediating its anti-aging effects. These effects suggest that QBLP alleviates aging likely through regulation of the gut microbiota structure and amino acid metabolism [25].

Further supporting the anti-aging potential of QBL-derived compounds, Thuraphan et al. (2024) identified a crude protein extract from QBL that strongly inhibited hyaluronidase activity, achieving an inhibition rate of 78.1 ± 1.5%, indicating a capacity to preserve extracellular matrix integrity—a hallmark of skin aging [23].

This accumulating evidence from invertebrate and mammalian models indicates that QBL possess substantial anti-aging properties. These effects appear to be mediated through multiple pathways, including enhanced antioxidant activity, modulation of longevity-related gene expression (e.g., via DAF-16), improvement of gut microbiota composition, and regulation of amino acid metabolism [25]. However, the specific bioactive components within QBL remain to be fully elucidated, warranting further purification and mechanistic studies.

### 5.2. Antioxidant Effects

Multiple lines of evidence from chemical and cell-based assays indicate the potent antioxidant properties of QBL extracts. For instance, Thuraphan et al. (2024) [23] reported that crude proteins and hydrolysates from QBL, especially when extracted with ascorbic acid, exhibited strong antioxidant and collagenase-inhibitory activities, comparable to those of ascorbic acid and lysine in ABTS and Griess assays. Importantly, these extracts caused no skin irritation in HET-CAM tests, suggesting their potential suitability for topical applications. Further supporting these findings, Haber et al. (2019) [30] evaluated the free radical scavenging capacity of QBL and pupae extracts under different dietary conditions using the 2,2-diphenyl-1-picrylhydrazyl (DPPH) method. They observed that larvae fed a mixed natural and sugar-water diet exhibited 21% free radical scavenging activity, while pupae derived from the same dietary regime showed 35% activity. Conversely, pupae reared exclusively on a natural diet exhibited approximately 48% antioxidant activity. Moreover, Sonmez et al. (2023) [52] compared the antioxidant capacities of DBL (drone bee larvae), QBL, and RJ via DPPH and ferric reducing antioxidant power (FRAP) assays. The half-maximal scavenging concentration (SC_50_) values for DPPH scavenging were 0.65 mg/mL for DBL and 0.84 mg/mL for QBL, markedly lower than that of RJ (7.94 mg/mL), indicating superior antioxidant potency. Tan Yao et al. (2019) [53] also highlighted the efficacy of a QBL-formulated cream, which showed 88.66% DPPH radical scavenging at 2.5 mg/mL, a performance equivalent to 0.1 mg/mL vitamin C.

In addition to direct free radical scavenging, QBL contain specific bioactive compounds that may contribute to the antioxidant effects. Yue Bing et al. (2010) [54] identified notable levels of taurine (3.35 mg/L) and coenzyme Q10 (20.08 mg/L) in enzymatically hydrolyzed QBL extracts, both of which are known to play critical roles in antioxidant defense, further confirming significant antioxidant activity. In vivo evidence reinforces these observations. Chen et al. (1997) [51] demonstrated that oral administration of RJ larvae to aged Wistar rats for 10 days significantly increased superoxide dismutase (SOD) activity and decreased MDA levels, indicating enhanced free radical clearance and reduced lipid peroxidation. Collectively, QBL exhibit considerable antioxidant properties across multiple experimental models, attributable to the direct free radical scavenging capacity.

### 5.3. Metabolic Regulation Effects

QBL also demonstrate potential metabolic regulatory activities. A study by Li (2023) [56] investigated the impact of dietary supplementation with lyophilized QBLP on Hy-Line Brown laying hens. The study revealed substantial enhancements in both production performance and metabolic parameters. The supplementation of QBLP led to enhanced production efficiency, as evidenced by a significant reduction in the feed-to-egg ratio, along with increased egg production rate and average egg weight. Moreover, egg quality parameters were notably improved, including eggshell strength and yolk color. Most notably, QBLP supplementation induced substantial metabolic alterations, manifesting as pronounced reductions in serum cholesterol levels by 14.15%, 16.60%, and 20.07% across different dosage groups [56]. These findings suggest that QBLP possesses bioactive components capable of modulating lipid metabolism and enhancing nutrient utilization efficiency in laying hens. The simultaneous improvement of production performance and metabolic profiles indicates that QBL may act through mechanisms that optimize energy allocation and metabolic homeostasis.

### 5.4. Other Bioactivities

QBL has been reported to ameliorate high-fat diet-induced obesity through the following mechanisms: by modulating gut microbiota composition and enhancing hepatic lipid metabolism while concurrently reducing systemic inflammation [12]. This metabolic benefit is complemented by substantial immunomodulatory effects. Earlier work showed that oral administration of RJ larvae significantly enhanced T-lymphocyte transformation, increased the thymus index, and elevated the carbon clearance index in mice indicators of improved cellular and non-specific immunity. The same treatment also markedly prolonged swimming endurance under varied temperature conditions, indicating enhanced stress resistance [58,59].

Further expanding the scope of bioactivities, lyophilized QBL powder alleviated p-chlorophenylalanine-induced insomnia in mice by modulating hypothalamic and serum levels of gamma-aminobutyric acid (GABA) and serotonin. This effect was accompanied by the restoration of gut microbiota composition, particularly an increase in Lactobacillus abundance, which was correlated with improved sleep-related neurotransmitter profiles [57]. Zhu (2007) [49] found that a chewable tablet formulated from QBL significantly enhanced exercise endurance in mice, prolonging swimming time, reducing blood lactic acid and serum urea nitrogen, increasing lactate dehydrogenase activity, and elevating hepatic glycogen reserves—collectively demonstrating strong anti-fatigue properties. Acute toxicity tests in the same study indicated a favorable safety profile. Taken together, the anti-obesity, immunomodulatory, anti-fatigue, and sleep-regulating effects attributed to QBL portray it as a systemic modulator of energy metabolism, immune function, and neuroendocrine homeostasis [12,52,58,59].

## 6. Conclusions and Prospects

This review synthesizes research on the nutritional composition of QBL, encompassing proteins, amino acids, fatty acids, and minerals. Concurrently, it examines studies on the bioactivities of QBL, including anti-aging, anti-inflammatory, and antioxidant properties. These findings provide a scientific basis for the potential utilization of QBL as a functional food source. The development of such applications can not only facilitate the development of novel nutrient-dense foods but also enhance global acceptance, promote the growth of the insect-based food industry, and unlock new opportunities within the food and health sector. Given recent scientific advances, several key research directions warrant further exploration from a food science perspective.

Firstly, the content of the chemical components in QBL remains heterogeneous with respect to origin and methodology, which limits cross-study comparability. Future study should focus on the standardized nutritional profiling of QBL across genetic strains, geographical origins, developmental stages, and rearing conditions. Harmonized analytical protocols, including explicit reporting of defatting status, nitrogen-to-protein conversion factors, and the treatment of chitin and other non-protein nitrogen fractions, are required to establish robust reference ranges for proteins, lipids, carbohydrates, and micronutrients. Such datasets will provide a reliable basis for the rational formulation of QBL-based products and for regulatory and nutritional assessment.

Secondly, the bioactivities and underlying mechanism of QBL-derived bioactive components need further studied. Proteins and their hydrolysates, specific peptides, lipids, and small molecules should be isolated and structurally characterized, followed by systematic evaluation of their roles in modulating oxidative stress, inflammation, aging-related signaling pathways, lipid metabolism, and gut microbiota. Dose–response relationships, bioavailability, and long-term safety must be clarified through well-designed animal studies and, where appropriate, early-phase human trials. Identifying which molecular fractions of QBL are responsible for specific biological effects will enable more targeted development of QBL-based nutraceuticals, cosmeceuticals, and functional foods.

Thirdly, from an industrial and translational perspective, processing and formulation technologies should be optimized to preserve or enhance the physicochemical properties and bioactivities of QBL while ensuring product stability, palatability and safety. This includes systematic comparison of drying, extraction, hydrolysis, and encapsulation strategies, as well as exploration of synergistic combinations of QBL with other hive products or probiotic ingredients. In parallel, comprehensive risk assessment, quality-control frameworks, and consumer acceptance studies will be essential to support the regulatory approval and successful market introduction of QBL-based products.

In summary, QBL currently stand at the interface between promising laboratory evidence and nascent industrial application. By integrating standardized compositional characterization, mechanism-oriented bioactivity research, and technology-driven product development, future studies can transform QBL from a underutilized by-product into a scientifically validated, high value-added resource for sustainable food systems and health-promoting products.

## Figures and Tables

**Table 1:** Nutritional composition of QBL (*Apis mellifera*).

Protein		Amino Acids	Fat	Ash	Total Carbohydrates	References
(Kjeldahl method)						
Kp 6.25	Kp 5.6					
—	22.10	—	19.8	4.0	54.1	[31]
55.80	—	46.67	18.64	6.32	19.24	[17]
51.35	—	—	—	4.95	17.48	[47]
48.70	—	37.30 (not defatted)	—	—	—	[23]
	—	39.72 (defatted)	—	—	—	
—	19.00	—	28.1	2.8	50.1	[30]
—	—	47.57	—	—	—	[45]
48.44	—	41.68	15.00	4.48	32.08	[21]
44.28	—	—	23.9	3.41	23.8	[48]
41.50	—	—	15.71	—	11.6	[49]
53.45	—	45.83	19.73	3.71	13.99	[10]

Note: Protein content determined by the Kjeldahl method is presented using dual conversion factors (Kp 6.25 and Kp 5.6), highlighting methodological differences in protein quantification. Values represent means reported for key components, including amino acids, fat, ash, and total carbohydrates. All nutritional values are expressed on a dry weight basis unless otherwise stated.

**Table 2:** Composition of amino acids in QBL under dry weight (*Apis mellifera*) [17,21].

Amino Acids	Content (g/100 g)
Aspartic Acid	5.48
Serine	1.66
Glutamic Acid	5.40
Glycine	2.09
Histidine	1.37
Arginine	2.25
Threonine	1.65
Alanine	2.22
Proline	2.01
Tyrosine	1.37
Valine	2.89
Methionine	0.84
Isoleucine	2.49
Leucine	3.45
Phenylalanine	2.07
Lysine	3.74
Tryptophan	0.44

**Table 3:** The health benefit bioactivity of QBL and components.

Biological Activity	Component	Experimental Model	References
Anti-oxidant	Acidic-extracted crude protein	In vitro (ABTS, Griess)	[23]
	Acidic-extracted protein hydrolysate		
	QBLP	C57BL/6J mice (D-gal-induced aging)	[25]
	QBLP	Naturally aged Wistar rats	[51]
	Freeze-Dried QBLP	In vitro assays (DPPH)	[30,52,53,54]
Anti-Aging	QBLP	*Musca domestica*	[55]
		NIH mice	
	QBLP	*Caenorhabditis elegans* (N2)	[24]
	Acidic-extracted crude protein	In vitro enzyme inhibition assay	[23]
	QBLP	C57BL/6J mice (D-gal-induced aging)	[25]
Metabolic Regulation	Freeze-Dried QBLP	Hy-Line Brown laying hens	[56]
Production Performance	Freeze-Dried QBLP	Hy-Line Brown laying hens	[56]
Anti-fatigue	QBLP	Kunming mice	[49]
Ameliorates obesity	Freeze-Dried QBLP	Diet-induced obese mice	[12]
Gut Microbiota Modulation	Freeze-Dried QBLP	C57BL/6J mice (D-gal-induced aging)	[25]
Anti-Insomnia	Freeze-Dried QBLP	C57BL/6 mice (PCPA-induced insomnia)	[57]

## Data Availability

No new data were created or analyzed in this study.
